# Short‐term exposure to synthetic flaxseed lignan LGM2605 alters gut microbiota in mice

**DOI:** 10.1002/mbo3.1185

**Published:** 2021-05-01

**Authors:** Reagan Badger, Ken Aho, Kinta Serve

**Affiliations:** ^1^ Department of Biological Sciences Idaho State University Pocatello ID USA

**Keywords:** dysbiosis, flaxseed, gut microbiota, LGM2605, systemic inflammation

## Abstract

LGM2605 is a synthetic version of the naturally occurring flaxseed lignan secoisolariciresinol diglucoside (SDG), with known anti‐inflammatory and antioxidant properties; however, its effects on gut microbial composition have not previously been evaluated. In the present study, we sought to determine how the 10‐day oral administration of LGM2605 alters the gut microbiota of mice. Eight‐week‐old female C57BL/6 mice were treated with either LGM2605 or saline, administered daily via oral gavage over a 10‐day treatment period. Upon termination of treatment, mouse cecums (*n* = 31) were collected, and cecal DNA was isolated. 16S rRNA genes were sequenced and analyzed in Mothur to identify changes in gut microbial composition induced by LGM2605 treatment (v. saline control). We then assessed community composition, performed indicator taxa analysis, and measured alpha and beta diversity. Overall, LGM2605 significantly altered the gut microbiota of mice; we reported alterations in 3 bacterial phyla and 22 genera as a result of treatment. The study here identifies for the first time significant alterations in the gut microbiota of mice following oral administration of LGM2605, in general shifting toward a more anti‐inflammatory composition. These findings lay the foundation for future investigations utilizing LGM2605 to control gut dysbiosis and, by extension, systemic inflammation.

## Graphical abstract

LGM2605 is a synthetic flaxseed lignan with both antioxidant and anti‐inflammatory properties. In the present study, we showed for the first time that short‐term exposure to LGM2605 beneficially alters the gut microbiota of mice. We found that orally administered LGM2605 may support a reduction of inflammation by increasing the growth of anti‐inflammatory bacteria while reducing the growth of pro‐inflammatory bacteria. Our findings support the therapeutic use of this compound in gut dysbiosis and other inflammatory disease models.

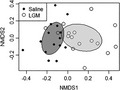

## 1 INTRODUCTION

In healthy individuals, the gut microbiome serves many vital functions: it aids in digestion, confers protection against pathogenic organisms, synthesizes essential vitamins and minerals, and plays an important role in the immune system (Clemente et al., 2018). Dysbiosis can cause dysregulation of these functions and has been linked to local and systemic inflammation as well as an autoimmune disease in both humans and animal models (Clemente et al., 2018). Factors contributing to dysbiosis and resulting inflammation include decreased overall bacterial diversity and overgrowth of more aggressive types of residential bacteria (Hakansson & Molin, 2011) For instance, a higher ratio of *Firmicutes*/*Bacteroidetes* in the gut is associated with increased body weight and increased systemic inflammation, while a lower *Firmicutes*/*Bacteroidetes* ratio supports the growth of beneficial bacteria and inhibits the growth of potential pathogens (Lin et al., 2019). Furthermore, dysbiosis may increase intestinal permeability or “leaky gut,” allowing for the passage of bacteria and toxins from the gut to the bloodstream or peritoneal cavity, where they promote inflammation and disease progression (Mu et al., 2017).

The dietary intervention is an effective means of improving gut health and ameliorating inflammatory responses by altering the diversity of gut microbes in ways that benefit, rather than disturb, host function (Clemente et al., 2018). Several previous studies have focused on dietary flaxseed supplementation in both human and animal subjects as a means of controlling inflammation and promoting healthy gut microbiota. Zhang et al. demonstrated in a 2017 study that administration of dietary flaxseed oil in mice ameliorated alcoholic liver disease by attenuating gut dysbiosis (decreasing the relative abundance of *Proteobacteria* and *Porphyromonadacea*), decreasing intestinal permeability, and reducing pro‐inflammatory cytokine levels (i.e., TNF‐α, IL‐1β, and IL‐6) in plasma (Zhang et al., 2017). Millman et al. further demonstrated in a 2019 study that dietary flaxseed oil administration in mice improved the overall gut microbial diversity and decreased the relative abundance of *Firmicutes*, outcomes that are typically associated with a reduction in systemic inflammation (Lin et al., 2019; Millman et al., 2020).

Despite the beneficial impacts of flaxseed consumption on gut microbiota, the utility of whole flaxseed as a dietary supplement is limited by its low bioavailability (Pietrofesa et al., 2016). Thus, researchers have turned to more readily metabolized flaxseed compound derivatives. Secoisolariciresinol diglucoside (SDG), a bioactive lignan highly concentrated in flaxseed, exhibits protective effects in a variety of systemic diseases, including cardiovascular and metabolic disorders (Kezimana et al., 2018). Several new studies also indicate protective effects of SDG in breast cancer with no adverse effects (Bowers et al., 2019; Delman et al., 2015; Fabian et al., 2020). The efficacy of SDG in a wide range of disease treatments is likely due to SDG’s antioxidant properties (Kezimana et al., 2018). Recently, the synthetic flaxseed lignan LGM2605 has been evaluated as a therapeutic alternative to naturally occurring SDG, as it has significantly greater bioavailability compared to whole flaxseed and can be readily produced in a laboratory setting (Mishra et al., 2013). Previous studies have demonstrated the usefulness of LGM2605 in controlling inflammation and oxidative stress responses in models of radiation and asbestos exposure, likely resulting from the ability of LGM2605 to scavenge radical species and reduce inflammatory cytokine production (Christofidou‐Solomidou et al., 2019; Mishra et al., 2020; Pietrofesa et al., 2014; Pietrofesa, Velalopoulou, Arguiri, et al., 2016; Velalopoulou et al., 2015). The protective effects of LGM2605 may be especially important in lung and breast cancer radiation therapies to reduce damage to bystander organs (Velalopoulou et al., 2017). Much like that of SDG, the biological activity of synthetic LGM2605 depends largely on metabolism by residential gut bacteria (Lin et al., 2019; Mishra et al., 2013). However, the influence of LGM2605 on gut microbial composition has not yet been evaluated.

In the present study, we sought to determine the effects of orally administered LGM2605 on the gut microbiota of mice. Identifying changes in gut microbiota will allow us to better understand the mechanism of action through which LGM2605 exerts its anti‐inflammatory effects, as the gut and immune system are inextricably linked. Previous studies have reported alterations in the murine gut microbiota as a result of dietary intervention in as little as 1 week (Wang et al., 2014). The human gut is generally less responsive to dietary intervention (Nguyen et al., 2015), but relatively minor compositional changes have been observed within just 10 days of treatment (Wu et al., 2011). Therefore, we selected a time frame of 10 days for the current study. Changes in gut microbial composition were identified by analysis of 16S rRNA genes of DNA extracted from mouse cecums. The cecum has been utilized in previous studies of gut microbiota in animals due to its high microbial diversity, which greatly exceeds that achieved by fecal sampling (Nguyen et al., 2015). We predicted that oral administration of LGM2605 would lead to beneficial changes in gut microbial composition relative to control, increasing the prevalence of bacteria characterized as anti‐inflammatory while decreasing the prevalence of bacteria characterized as pro‐inflammatory.

## 2 MATERIALS AND METHODS

### 2.1 Animals and diet

Forty‐eight female C57BL/6 mice (8 weeks old) were obtained from Jackson Laboratories. This age was chosen as mice are mature with relatively stable gut microbiota (Korach‐Rechtman et al., 2019; Laukens et al., 2016). The animals were housed 4/cage in the Idaho State University Animal Care Facility, with a 12‐h light/dark cycle, constant temperature (22°C), and constant humidity (45%). Throughout the study, mice were given *ad libitum* access to standard rodent chow (LabDiet 5V5R) and filtered water. Mice were allowed to acclimate for 6 days preceding administration of treatment.

### 2.2 LGM2605 treatment

Synthetic SDG (referred to as LGM2605 in the literature) was independently generated as previously described (Mishra et al., 2013). Briefly, LGM2605 was synthesized from vanillin via secoisolariciresinol and glucosyl donor (perbenzoyl‐protected trichloacetimidate under the influence of TMSOTf) through a concise route involving chromatographic separation of diastereomeric diglucoside derivatives (Chemveda Life Sciences, Inc.). Lyophilized samples of LGM2605 (100 mg/vial) were reconstituted with sterile, endotoxin‐free water to produce a stock solution of 50 mg/mL. Endotoxin testing of water was performed before the preparation of stock solution using the ToxinSensor^TM^ Chromogenic LAL Endotoxin Assay Kit (GenScript Biotech Corp), following the manufacturer's protocol. Water samples were tested against a standard curve provided with the kit. Briefly, the samples were mixed with 100µL of LAL, added to endotoxin‐free tubes, and incubated at 37°C for 6 min. Stop solutions and color stabilizers were added to each tube, and then 200µL of each solution was analyzed by measuring absorbance at 545 nm in triplicate for each sample. Endotoxin was detected at 0.59EU/mL, which is below the acceptable lower limit for oral administration in mice. LGM2605 (100 mg/kg body weight) was administered daily using curved 2‐inch 18‐gauge stainless steel feeding tubes (sterilized before use). Individual mouse body weights were measured daily to calculate appropriate dosage volumes. This dosage was experimentally determined in previous studies (Christofidou‐Solomidou et al., 2012; Pietrofesa, Velalopoulou, Arguiri, et al., 2016). Control animals received an equivalent volume of saline via oral gavage. Mice were randomly assigned to either the experimental group (LGM2605) or the control group (saline); treatments were administered for a total of 10 days.

### 2.3 Cecal DNA extraction and 16S rRNA sequencing

Following the 10‐day treatment period, mice were euthanized by CO_2_ asphyxiation. A total of 31 cecum samples were collected into sterile microfuge tubes and frozen at −20°C until needed for analysis. Cecums were then thawed at room temperature, and cecal DNA was extracted using the Qiagen QIAamp^TM^ PowerFecal^TM^ DNA Kit. Briefly, each cecum was sectioned into two pieces of approximately equal size, and one section was transferred to a Dry Bead Tube provided in the kit (up to 0.25 g biosolid/tube). The second section was re‐frozen for later use. Subsequent steps were performed according to the manufacturer's instructions. The bead‐beating step was performed using the Mini‐Beadbeater‐8 (BioSpec). DNA was eluted in 100µL of C6 elution buffer solution. Recovered DNA quantity and quality were assessed using the NanoDrop ND‐1000 spectrophotometer (Marshall Scientific). Cecal DNA was then amplified and sequenced by the Molecular Core Research Facility (MRCF) of Idaho State University. Briefly, variable region 4 (V4) of bacterial 16S ribosomal RNA genes was amplified by PCR, and subsequent cleanup was performed. Library integrity was assessed by running a portion of the samples through AATI Fragment Analysis and qPCR for quality control. Samples were then quantified with the Qubit 2.0 Fluorometer and pooled in equimolar amounts. The sample pool was sequenced on an Illumina MiSeq platform using a 2x250‐bp Miseq Reagent Kit v3 (Illumina). Sequencing of the 31 samples yielded a total of 6,256,091 reads, with a mean read count of 201,809 and a range of 37,544–428,248 reads. Amplicon sequence data was processed in Mothur. Sequences were clustered into operational taxonomic units (OTUs) at 97% identity using SILVA Taxonomy; a total of 9,914 unique OTUs were identified.

### 2.4 Statistical analyses

The effects of LGM2605 treatment on gut microbial composition were investigated primarily using analysis methods in Phyloseq (R Bioconductor package) (McMurdie & Holmes, 2013). PERMANOVA (Anderson, 2001) was used to test for significant differences in the composition of bacterial communities resulting from LGM2605 treatment, relative to control. Compositional patterns of communities were visually depicted using non‐metric multidimensional scaling (NMDS) (Kruskal, 1964). The indicator taxa analysis of Dufrene–Legendre (Pietrofesa, Velalopoulou, Arguiri, et al., 2016) was used to determine which groups of bacteria exhibited significant differences in abundance as a result of treatment, assessing both phylum‐ and genus‐level comparisons. We used the nonparametric *indval* function from the package labdsv (CRAN, 2019) to derive indicator values and to determine statistical significance. Measures of alpha and beta diversity were also calculated for each treatment group. Shannon's and Simpson's diversity indices were used to calculate alpha diversity; the Bray–Curtis dissimilarity index (Bray & Curtis, 1957) was used to calculate beta diversity. Beta diversity was partitioned into balanced variation and abundance gradients using the *beta*.*pair*.*abund* function from the package betapart (Baselga, 2013). Differences in beta diversity with respect to treatment were assessed using Anderson's PERMDISP2 procedure (Anderson, 2006), which provides an analysis of multivariate homogeneity of group variances.

## 3 RESULTS

### 3.1 LGM2605 treatment was shown to significantly alter gut microbial composition in mice

**FIGURE 1:**
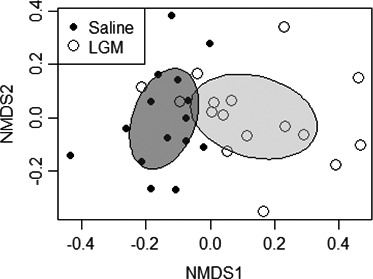
NMDS plot. Ordination is based on Bray–Curtis dissimilarity matrix generated from the operational taxonomic unit (OTU) table of sequencing data. Points represent individual cecum samples (*n* = 31); distance between points represents the level of similarity between them. The plot demonstrates that bacterial communities differ between cecums of mice treated with LGM2605 v. cecums of mice treated with saline only (*p* < 0.001 by PERMANOVA)

**FIGURE 2:**
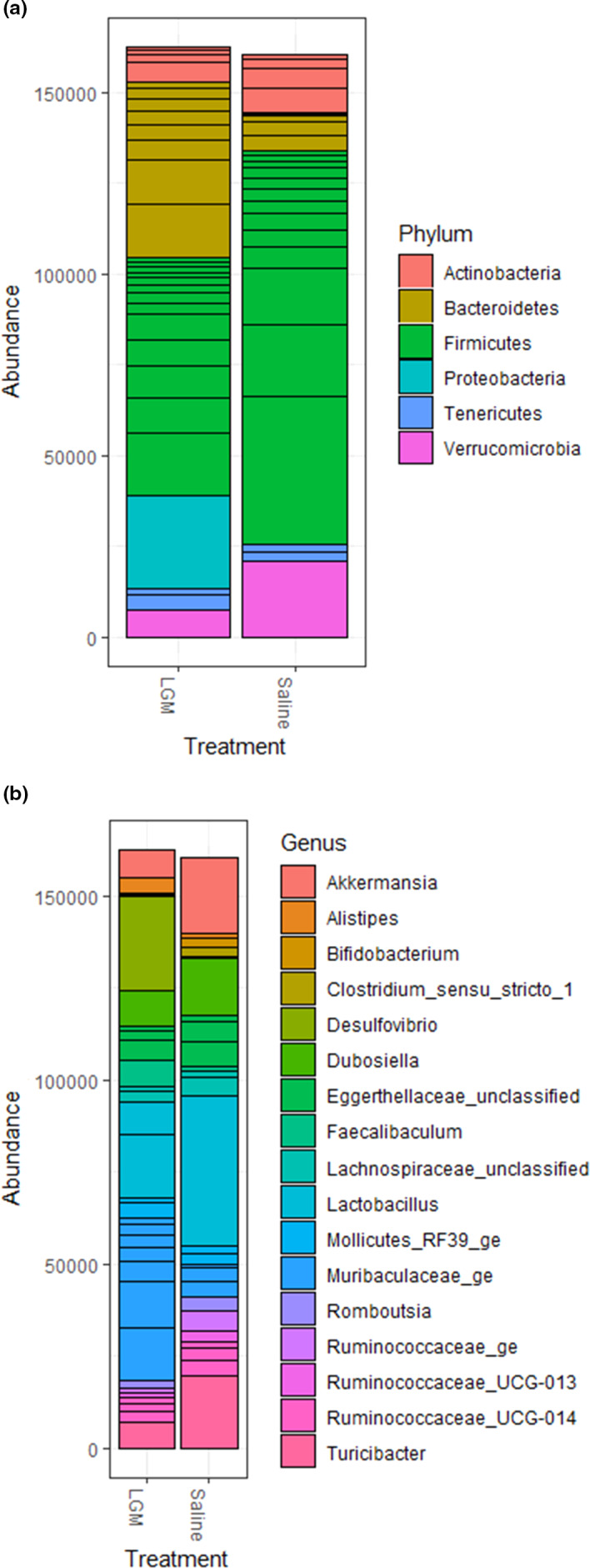
Stacked bar plots of bacterial phylogenetic distribution, by phylum (a) and by genus (b). Classifications were assigned using SILVA Taxonomy. OTUs were averaged among individual cecum samples (*n* = 31) so that group differences in relative bacterial abundance could be compared. The plots demonstrate that gut microbial composition differs at both the phylum and genus levels between cecums of mice treated with LGM2605 v. cecums of mice treated with saline only

Oral administration of LGM2605 significantly altered gut microbial composition as compared to saline only (*p *< 0.001 by PERMANOVA). The NMDS plot (Figure 1) illustrates the level of similarity between constituent bacterial communities in cecal samples, comparing cecums of LGM2605‐treated v. saline‐treated mice. Stacked bar plots illustrate bacterial phylogenetic distribution by phylum (Figure 2a) and by genus (Figure 2b), again comparing cecums of LGM2605‐treated v. saline‐treated mice.

### 3.2 LGM2605 treatment altered the total abundance of 3 bacterial phyla and 22 genera compared to saline only

Indicator taxa analysis revealed significant differences in bacterial abundance across 3 phyla as a result of LGM2605 treatment (Table 1). Notably, LGM2605 treatment decreased the *Firmicutes*/*Bacteroidetes* ratio relative to saline only. In addition, LGM2605 treatment significantly altered 22 bacterial genera compared to saline (Table 2). Of those identified, 7 genera were clearly defined in scientific literature as having pro‐ or anti‐inflammatory activity, as described in the discussion.

**TABLE 1:** Results of indicator taxa analysis, phylum level

Indicator taxa analysis: Effects of LGM2605 on Bacterial phyla abundance						
Phylum	Median # Reads ±IQR		Indicator Value		Effects of LGM2605 on Bacterial Abundance	*p*‐Value
	Saline	LGM2605	Saline	LGM2605		
*Proteobacteria*	0.171 ± 0.278	14.528 ± 69.149	0.009	0.991	Increased	0.001
*Bacteroidetes*	3.263 ± 4.528	20.835 ± 24.634	0.200	0.800	Increased	0.003
*Firmicutes*	22.751 ± 10.981	17.314 ± 18.320	0.392	0.608	Decreased	0.026

Note

Indicator values account for fidelity and relative abundance and range from 0 to 1, with higher values for stronger indicators. Significant differences in bacterial abundance as a result of LGM2605 treatment (relative to saline only) were observed across 3 phyla within cecal samples (*n* = 31). Table reports increased/decreased relative abundance in response to LGM2605 treatment. Phyla that were not significantly altered by LGM2605 treatment are not reported.

**TABLE 2:** Results of indicator taxa analysis, genus level

Indicator taxa analysis: Effects of LGM2605 on Bacterial genera abundance						
Genus	Median # Reads ±IQR		Indicator value		Effects of LGM2605 on bacterial abundance	*p*‐value
	Saline	LGM2605	Saline	LGM2605		
*Alistipes* ^*^	9.081 ± 6.346	23.477 ± 16.911	0.247	0.753	Increased	0.001
*Desulfovibrio* ^*^	0.058 ± 0.080	22.910 ± 110.265	0.002	0.998	Increased	0.001
*Desulfovibrionaceae_unclassified*	0.000 ± 0.000	0.031 ± 0.107	0.002	0.617	Increased	0.001
*Muribaculaceae_ge*	4.980 ± 8.131	38.340 ± 44.343	0.193	0.807	Increased	0.002
*Bacteroidales_unclassified*	0.015 ± 0.027	0.058 ± 0.085	0.129	0.706	Increased	0.005
*Anaeroplasma* ^*^	0.571 ± 3.071	7.857 ± 16.536	0.027	0.903	Increased	0.009
*Faecalibaculum* ^*^	0.651 ± 1.805	18.251 ± 87.097	0.148	0.852	Increased	0.010
*Ruminoclostridium_5*	3.594 ± 9.422	12.563 ± 15.305	0.233	0.767	Increased	0.010
*GCA−900066225*	7.000 ± 15.750	19.500 ± 10.750	0.256	0.704	Increased	0.019
*Muribaculaceae_unclassified*	0.010 ± 0.018	0.049 ± 0.067	0.234	0.684	Increased	0.048
*Candidatus_Stoquefichus*	14.667 ± 19.333	5.833 ± 4.417	0.803	0.198	Decreased	0.001
*Bifidobacterium* ^*^	34.672 ± 47.141	4.289 ± 12.574	0.798	0.202	Decreased	0.002
*Lachnospiracea_UCG−001*	2.963 ± 26.500	0.315 ± 1.361	0.941	0.052	Decreased	0.005
*Ruminococcaceae_UCG−013*	16.913 ± 25.245	9.121 ± 11.404	0.733	0.267	Decreased	0.008
*Turicibacter* ^*^	26.601 ± 22.732	11.847 ± 9.455	0.737	0.263	Decreased	0.010
*Clostridium_sensu_stricto_1*	37.100 ± 228.900	17.300 ± 29.425	0.916	0.084	Decreased	0.011
*Marvinbryantia*	3.250 ± 17.875	0.500 ± 1.625	0.859	0.060	Decreased	0.011
*Ruminococcaceae_ge*	9.697 ± 57.616	4.130 ± 7.689	0.795	0.205	Decreased	0.020
*Streptococcus* ^*^	20.200 ± 15.500	9.900 ± 16.650	0.737	0.263	Decreased	0.026
*Clostridiacea_1_unclassified*	0.015 ± 0.045	0.000 ± 0.015	0.593	0.041	Decreased	0.035
*Erysipelatoclostridium*	21.900 ± 17.250	1.050 ± 11.950	0.726	0.257	Decreased	0.038
*Lachnospiraceae_unclassified*	14.415 ± 11.407	9.099 ± 5.995	0.611	0.389	Decreased	0.047

Note

Indicator values account for fidelity and relative abundance and range from 0 to 1, with higher values for stronger indicators. Significant differences in bacterial abundance as a result of LGM2605 treatment (relative to saline only) were observed across 22 genera within cecal samples (*n* = 31). Table reports increased/decreased relative abundance in response to LGM2605 treatment. Genera that were not significantly altered by LGM2605 treatment are not reported.

* Of these, 7 genera (indicated by asterisks) were identified in the literature as playing important roles in inflammation and/or autoimmune disease.

### 3.3 LGM2605 treatment did not impact alpha diversity of gut microbiota

**FIGURE 3:**
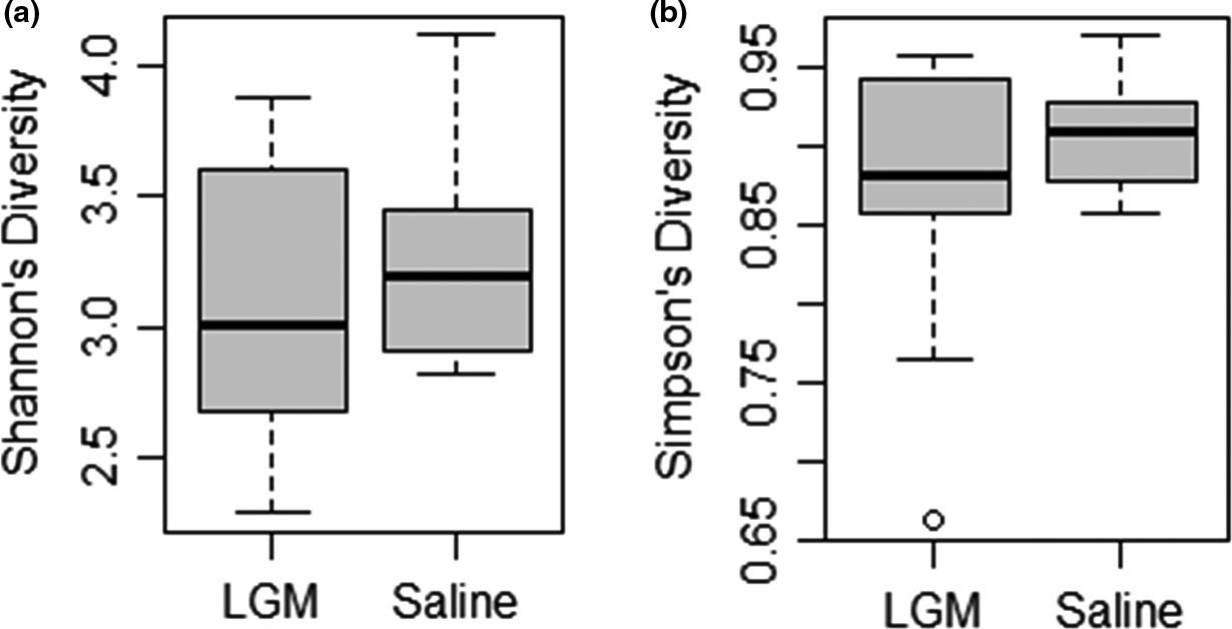
Box plots of Shannon's diversity (a) and Simpson's diversity (b), quantifying overall biodiversity within individual cecum samples (*n* = 31). Shannon's diversity is reported as H; Simpson's diversity is reported as D. Greater richness and evenness of bacterial communities correspond with higher diversity measures. For LGM2605‐treated mice, H=3.766363 and D=0.9531014; for saline‐treated mice, H=3.564396 and D=0.9287959. Based on these measures, we report no significant difference in the level of alpha diversity observed in cecums of LGM2605‐treated v. saline‐treated mice.

Alpha diversity was calculated using both Shannon's diversity index (H) and Simpson's diversity index (D). These indices reflect the richness and evenness of bacterial communities (OTUs) in individual cecal samples. For LGM2605‐treated mice, H = 3.766 and D = 0.953; for saline‐treated mice, H = 3.564 and D = 0.929. Box plots illustrate alpha diversity of gut microbiota by treatment group (Figure 3). Both LGM2605‐treated and saline‐treated mice exhibited high alpha diversity in cecal samples, but no significant difference in alpha diversity by either Shannon's or Simpson's method was observed as a result of LGM2605 treatment.

### 3.4 LGM2605 treatment did not impact beta diversity of gut microbiota

Beta diversity was calculated using the Bray–Curtis dissimilarity index, characterizing abundance‐based dissimilarity between cecal samples. For LGM2605‐treated mice, total beta diversity = 0.657; for saline‐treated mice, total beta diversity = 0.670. Among both treatment groups, beta diversity was primarily attributed to balanced variation in bacterial abundance (balanced variation = 0.478, LGM2605; 0.491, saline) rather than unidirectional abundance gradients (abundance gradients = 0.180, LGM2605; 0.179, saline). No significant difference in beta diversity was observed as a result of LGM2605 treatment using the PERMDISP2 procedure.

## 4 DISCUSSION

The gut microbiota and systemic inflammation are inextricably linked, as emerging research has demonstrated (Clemente et al., 2018; Hakansson & Molin, 2011; Lin et al., 2019; Mu et al., 2017). Dietary intervention may help to beneficially alter the gut microbial composition, producing anti‐inflammatory effects. In the present study, we utilized the synthetic flaxseed lignan LGM2605 as a form of dietary intervention in mice. Previous studies have shown that LGM2605 acts as an antioxidant and free radical scavenger (Pietrofesa, Velalopoulou, Arguiri, et al., 2016), reducing inflammation associated with radiation and asbestos exposure (Christofidou‐Solomidou et al., 2019; Mishra et al., 2020; Velalopoulou et al., 2015); however, its effects on gut microbiota have not previously been evaluated. We therefore sought to determine how a 10‐day period of oral LGM2605 administration affects the gut microbial composition of mice. We utilized female mice for this study since SDG is of particular interest for breast cancer therapies (Bowers et al., 2019; Delman et al., 2015; Fabian et al., 2020) and analyzed 16S rRNA extracted from mouse cecums. The cecum is proportionately larger in mice as compared to humans due to its importance in the digestion of plant compounds within the mouse gut; thus, it is not a perfect homolog for humans (Hugenholtz & Vos, 2018). However, the mouse cecum is still a valid model for human health associations because major bacterial community composition is conserved among mammals (Nguyen et al., 2015). Additionally, the cecum is a preferred site of DNA sequencing and more representative than fecal sampling based on the high microbial diversity in this intestinal region (Nguyen et al., 2015); it was therefore selected for analysis in this study.

LGM2605 treatment led to significant alterations in gut microbial composition compared with saline only (*p *< 0.001 by PERMANOVA). Subsequent analyses were performed to evaluate the implications of these changes for systemic inflammation and human health. In general, we predicted that the gut‐modulating effects of synthetic LGM2605 would mirror those of orally administered flaxseed oil, as observed in previous studies (Millman et al., 2020; Zhang et al., 2017). We hypothesized that oral administration of LGM2605 would increase the relative abundance of “beneficial,” or anti‐inflammatory, gut bacteria as well as decrease the relative abundance of bacteria linked to inflammation and autoimmunity.

At the phylum level, LGM2605 treatment led to a decreased *Firmicutes*/*Bacteroidetes* ratio (Table 1), which is generally associated with lower intestinal and systemic inflammation, lower risk of obesity, and higher overall bacterial diversity (Verdam et al., 2013). However, LGM2605 treatment also led to an increased prevalence of *Proteobacteria*, in contrast to the 2017 study by Zhang et al., (2017), which reported a decrease in *Proteobacteria* as a result of dietary flaxseed oil supplementation. Because *Proteobacteria* is often a marker of intestinal dysbiosis (Shin et al., 2015), further research is needed to understand what factors may have contributed to this effect in LGM2605‐treated mice and the potential health implications.

At the genus level, LGM2605 treatment contributed to an increase in several genera associated with anti‐inflammatory effects, including *Alistipes*, *Anaeroplasma*, and *Faecalibaculum* (Table 2). Both *Alistipes* and *Anaeroplasma* are associated with increased production of the anti‐inflammatory cytokine TGF‐β, which upregulates mucosal IgA expression, thereby strengthening the intestinal barrier and reducing gut permeability (Beller et al., 2020; Parker et al., 2020). *Alistipes* is also associated with increased production of the anti‐inflammatory cytokine IL‐10, which may help to suppress overactive immune responses (Parker et al., 2020). During digestion, both *Alistipes* and *Faecalibaculum* produce short‐chain fatty acids (SCFAs), bacterial metabolites that have been shown to improve intestinal barrier function (Han et al., 2019; Parker et al., 2020). SCFAs help suppress Th‐17 cells and support differentiation of T‐regulatory cells (T‐regs), which is crucial in maintaining gut homeostasis and combatting inflammation (Opazo et al., 2018). The *Faecalibaculum* genus includes only one species, *Faecalibaculum rodentium*, which is found exclusively in mice; its human homolog is *Holdemanella biformis*. Studies of intestinal tumorigenesis have shown that *F. rodentium* and *H. biformis* may possess both anti‐inflammatory and anti‐tumorigenic effects through the production of SCFAs (Zagato et al., 2020).

LGM2605 treatment also contributed to a decrease in several genera that are associated with pro‐inflammatory effects, including *Turicibacter* and *Streptococcus* (Table 2). Both of these genera are implicated in various forms of autoimmune disease, including inflammatory bowel disease and rheumatoid arthritis (Bernstein & Forbes, 2017; Heidarian et al., 2019). *Streptococcus* induces expression of the pro‐inflammatory cytokines TNF‐α, IL‐6, and IFN‐γ, which are associated with autoimmunity (Jiang et al., 2015). The role of *Turicibacter* in disease pathology remains largely uncharacterized, but some studies report that its presence may be related to TNF expression (Bernstein & Forbes, 2017).

Interestingly, LGM2605 treatment led to an increased abundance of *Desulfovibrio*, which is generally regarded as pro‐inflammatory. *Desulfovibrio* is a genus of gram‐negative bacteria that produces lipid A, the toxic subunit of lipopolysaccharides (LPS) (Zhang‐Sun et al., 2015). In the case of “leaky gut,” LPS translocates from the intestines to the bloodstream, where it stimulates pro‐inflammatory cytokine release (e.g., TNF‐α, IL‐6, IL‐1) in macrophages, contributing to systemic inflammation (Hakansson & Molin, 2011). Zhang et al. reported decreased plasma LPS following flaxseed oil supplementation; while not measured within the current study, plasma LPS may serve as a more accurate representation of LPS translocation and resulting inflammation (Zhang et al., 2017). Taken together, our findings suggest that the increased *Desulfovibrio* abundance following LGM2605 treatment was either insufficient to affect the gut lumen or was overridden by other microbial alterations, such as the increased prevalence of anti‐inflammatory bacteria. More research is needed to tease apart this complicated relationship and to more fully understand the effects of *Desulfovibrio* on systemic inflammation.

LGM2605 treatment also led to a decreased abundance of *Bifidobacterium*, which is considered to be both anti‐inflammatory and probiotic (Table 2). As a probiotic, *Bifidobacterium* alters gut microbial composition by stimulating the growth of residential bacteria while reducing the growth of bacterial pathogens. Furthermore, *Bifidobacterium* may help to regulate the balance of T‐helper 1 (Th1)/T‐helper 2 (Th2) cells. Th1‐biased immune responses are often implicated in autoimmune disease and increased production of the pro‐inflammatory cytokines IFN‐γ and TNF‐α. Restoring the balance of Th1/Th2 cells may therefore be beneficial in disease prevention (Medina et al., 2008). *Bifidobacterium* has been shown to suppress pro‐inflammatory cytokine production as well as improve intestinal barrier function via this mechanism (Lobionda et al., 2019; Medina et al., 2008). The effects of LGM2605 on *Bifidobacterium* abundance and their associated health implications may be another consideration for future research.

Effects of dietary intervention on gut microbial composition are conventionally measured not only through the response of individual bacterial communities but also through alterations in the overall biodiversity of gut microbiota, that is, alpha diversity. Decreased alpha diversity has been reported in various forms of autoimmune disease, including inflammatory bowel disease, rheumatoid arthritis (Bernstein & Forbes, 2017), and systemic lupus erythematosus (Khan & Wang, 2019). Although LGM2605 treatment significantly altered the bacterial abundance of multiple phyla/genera within cecal samples (Table 2), it did not impact alpha diversity measures by either Shannon's or Simpson's method (Figure 3). The lack of change in overall gut biodiversity may be partially explained by the short study duration of 10 days, as opposed to 6 or more weeks, which is the experimental length of other similar dietary studies. However, LGM2605 treatment still contributed to significant changes in gut microbial composition within just 10 days, providing compelling evidence for future studies investigating the effects of longer‐term treatment.

Additionally, we found that LGM2605 treatment did not impact beta diversity measures, which describe the heterogeneity in microbial composition between cecums, that is, inter‐sample variation. Among both treatment groups, beta diversity was driven primarily by balanced variation, or variation in the prevalence of specific bacterial communities from one sample to the next, as opposed to variation in the number of individual organisms detected (Baselga, 2013). Future studies may seek to determine the inflammatory effects of individual genera and/or species by colonizing the GI tracts of germ‐free mice with microorganisms of interest. This approach would allow for the differentiation of specific bacterial communities and their respective roles in disease, independent of other physiologic influences (Khan & Wang, 2019). However, differences observed should also be studied collectively in consideration of their combined effects on inflammation and disease pathology.

Since this study focused on the effects of short‐term LGM2605 treatment, we were unable to fully evaluate the compound's ability to modulate gut microbiota or systemic inflammation. However, the alterations in community composition that we observed—including changes in the relative abundance of 3 bacterial phyla as well as 22 genera—are encouraging and evoke the need for future studies investigating the effects of longer‐term LGM2605 treatment. Future studies should also consider the application of LGM2605 in gut dysbiosis models, to further establish the ability of LGM2605 to beneficially alter gut microbial composition. LGM2605 has previously demonstrated protective effects in the context of radiation and asbestos exposure, which are known inducers of inflammation (Bowers et al., 2019). While these effects have been largely attributed to the compound's direct antioxidant and free radical scavenging abilities, its gut‐modulating effects may also play a role in reducing systemic inflammation within these models. Finally, the present study was limited by its exclusive use of 16S rRNA gene sequencing; future studies should also utilize transcriptomics and/or metabolomics to more fully elucidate the functional responses of gut microbes to LGM2605.

Overall, this study provides evidence that short‐term dietary treatment with the synthetic flaxseed derivative LGM2605 significantly alters the gut microbiota of mice, in general shifting towards a more anti‐inflammatory composition. Despite the short experimental duration, the findings presented here suggest that LGM2605 treatment positively impacts the gut microbiota of mice, which may contribute to the previously reported anti‐inflammatory, antioxidant, and chemoprotective effects of LGM2605 (Pietrofesa Solomides, & Christofidou‐Solomidou, 2014; Pietrofesa, Velalopoulou, Arguiri, et al., 2016; Pietrofesa et al., 2017; Velalopoulou et al., 2015). This study also establishes baseline changes in gut microbiota following oral LGM2605 administration, which will be an important reference point for future studies.

## CONFLICTS OF INTEREST

None declared.

## AUTHOR CONTRIBUTIONS

**Reagan Badger:** Conceptualization (equal); Data curation (lead); Formal analysis (lead); Investigation (equal); Validation (equal); Writing‐original draft (lead); Writing‐review & editing (lead). **Ken Aho:** Formal analysis (equal); Methodology (supporting); Software (supporting); Validation (supporting); Writing‐review & editing (supporting). **Kinta Serve:** Conceptualization (lead); Data curation (supporting); Funding acquisition (lead); Investigation (equal); Methodology (supporting); Project administration (supporting); Resources (lead); Supervision (supporting); Writing‐original draft (supporting); Writing‐review & editing (supporting).

## ETHICS STATEMENT

All experiments were conducted following the approval granted by the Idaho State University Institutional Animal Care and Use Committee (IACUC).

## Data Availability

All data are provided in full in this manuscript aside from 16S rRNA sequence reads. Sequence data are available at https://www.mg‐rast.org/linkin.cgi?project=mgp98134.
